# Supplementary material for: Indirect CRISPR screening with photoconversion revealed key factors of drug resistance with cell–cell interactions
Source: Commun Biol. 2023 Jun 1;6:582. doi: 10.1038/s42003-023-04941-9 (PMC10235018; doi:10.1038/s42003-023-04941-9)
Supplement: Supplementary file 2 — Supplementary Information [file 42003_2023_4941_MOESM2_ESM.pdf]

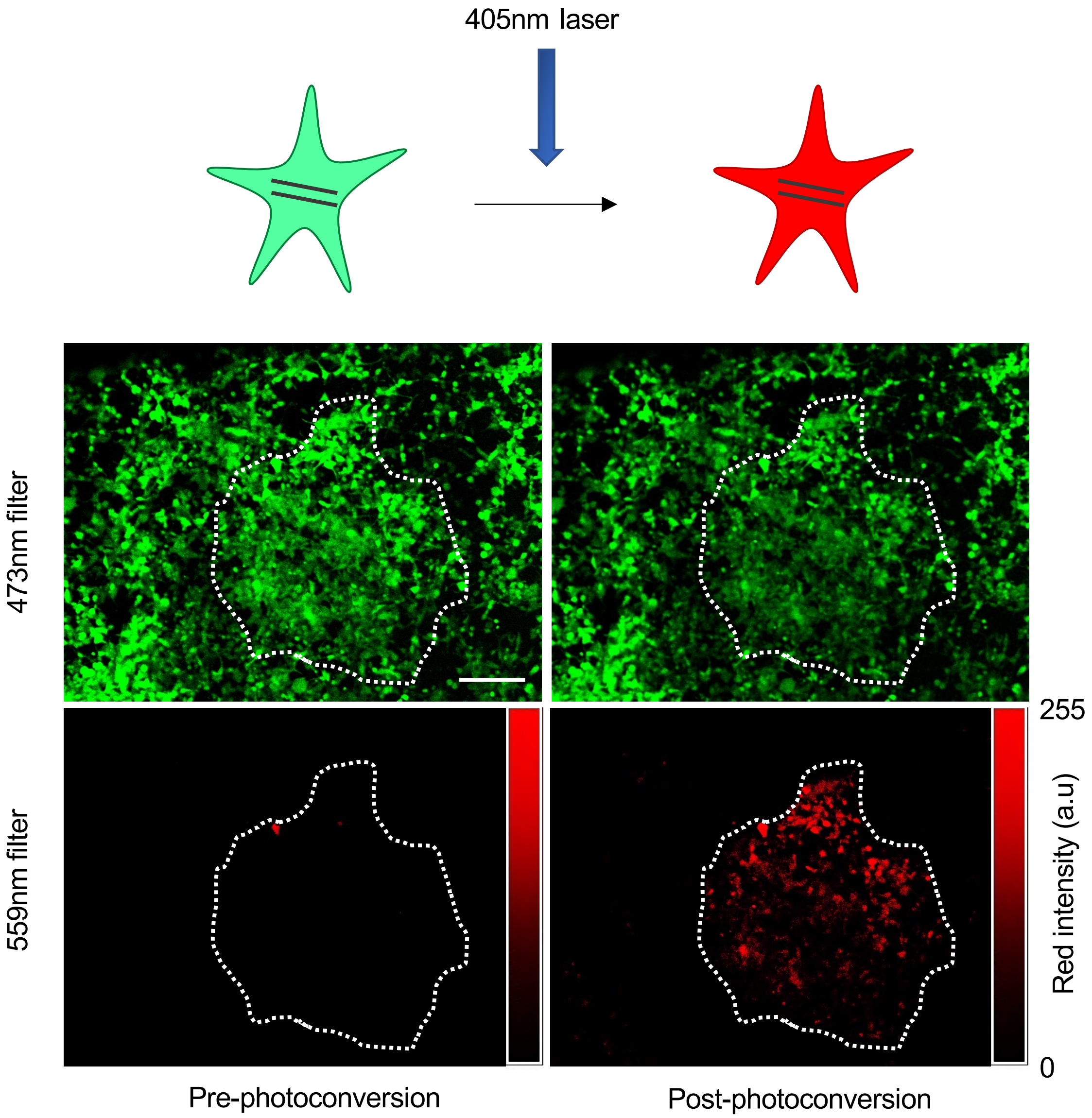

Supplementary Figure 1. Dendra2 photoconversion. Dendra2 is a green fluorescent protein, and illumination with the 405 nm UV laser can irreversibly convert its fluorescence wavelength from green to red. The lower panel shows photographs of the actual cells pre- and post-photoconversion, in which red fluorescence was induced by illuminating the dotted line with the 405 nm laser. Image captures of the green form of Dendra2 were performed with the 473 nm laser (upper tier), whereas the red form was performed with the 559 nm laser (lower tier). Scale bar, 50 μm.

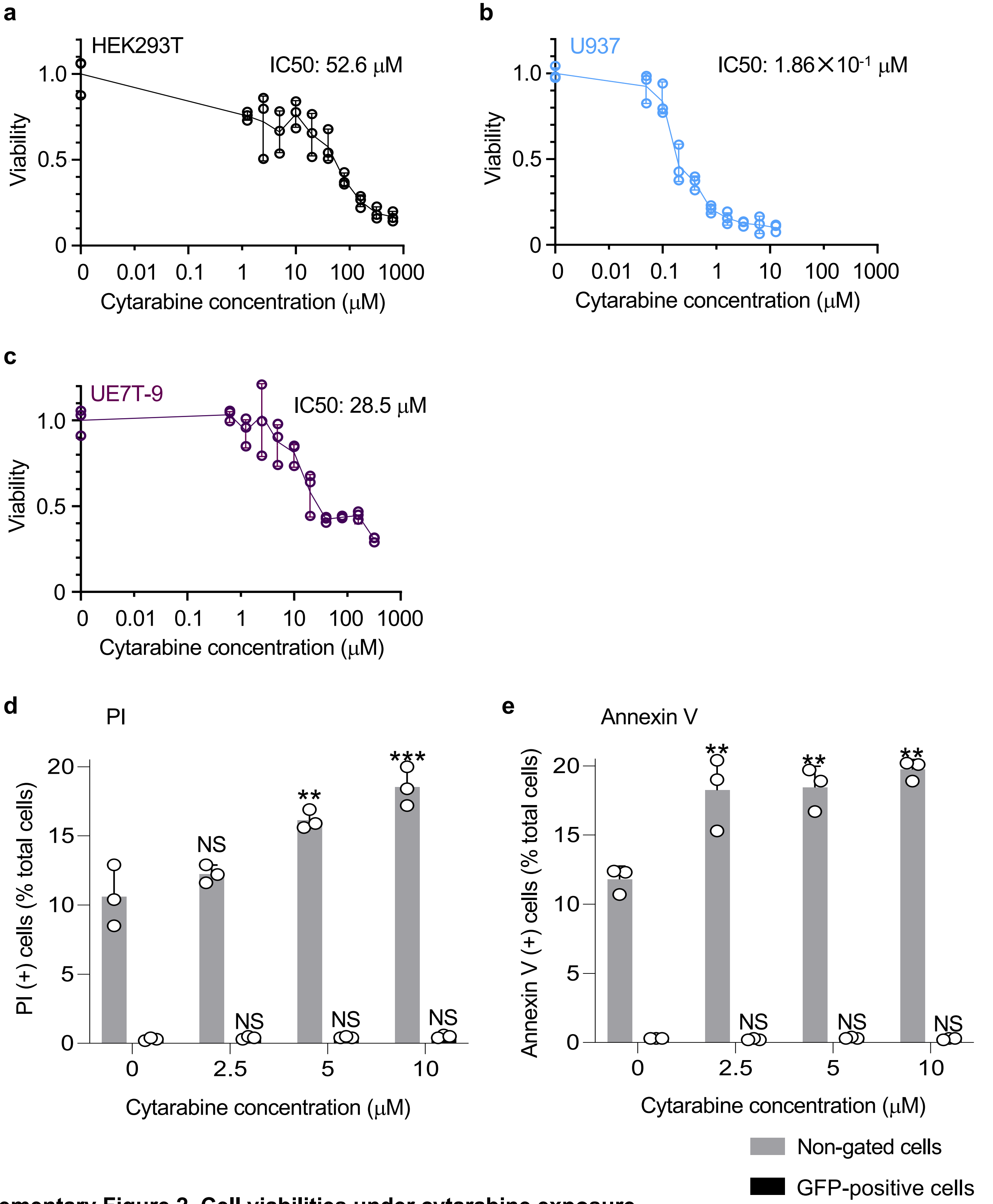

Supplementary Figure 2. Cell viabilities under cytarabine exposure.
(a-c) Dose-response curves of HEK293T (**a**), U937 (**b**), and UE7T-9 (**c**) cells treated with cytarabine for 48 h. IC50 to cytarabine are shown in each graph. **(d, e)** GFP-positive cells represent viable U937 cells. In the co-culture system with HEK293T cells, GFP-positive U937 cells were exposed to cytarabine. After 48 h of exposure to cytarabine, Propidium iodide (PI) (**d**) or Annexin V (**e**) was evaluated. Under the non-gated condition, PI-positive (**d**) or Annexin V-positive (**e**) cells increased with cytarabine exposure. However, under the GFP-gated condition, few PI (**d**) or Annexin V (**e**) positive cells were observed at any cytarabine concentration. So, almost all PI-positive or Annexin V-positive cells do not express GFP, and therefore, GFP-positive U937 cells represent living U937 cells. The experiment was performed with biological triplication in the two independent experiments. Data are represented as mean ± SD. Statistical significance values were calculated by performing one-way ANOVA with Dunnett's test (**d, e**). In the analysis of PI or Annexin V (**d, e**), the control was "cytarabine 0 μM" of each gating condition. **$p < 0.01$, ***$p < 0.001$, and NS: non-significant.

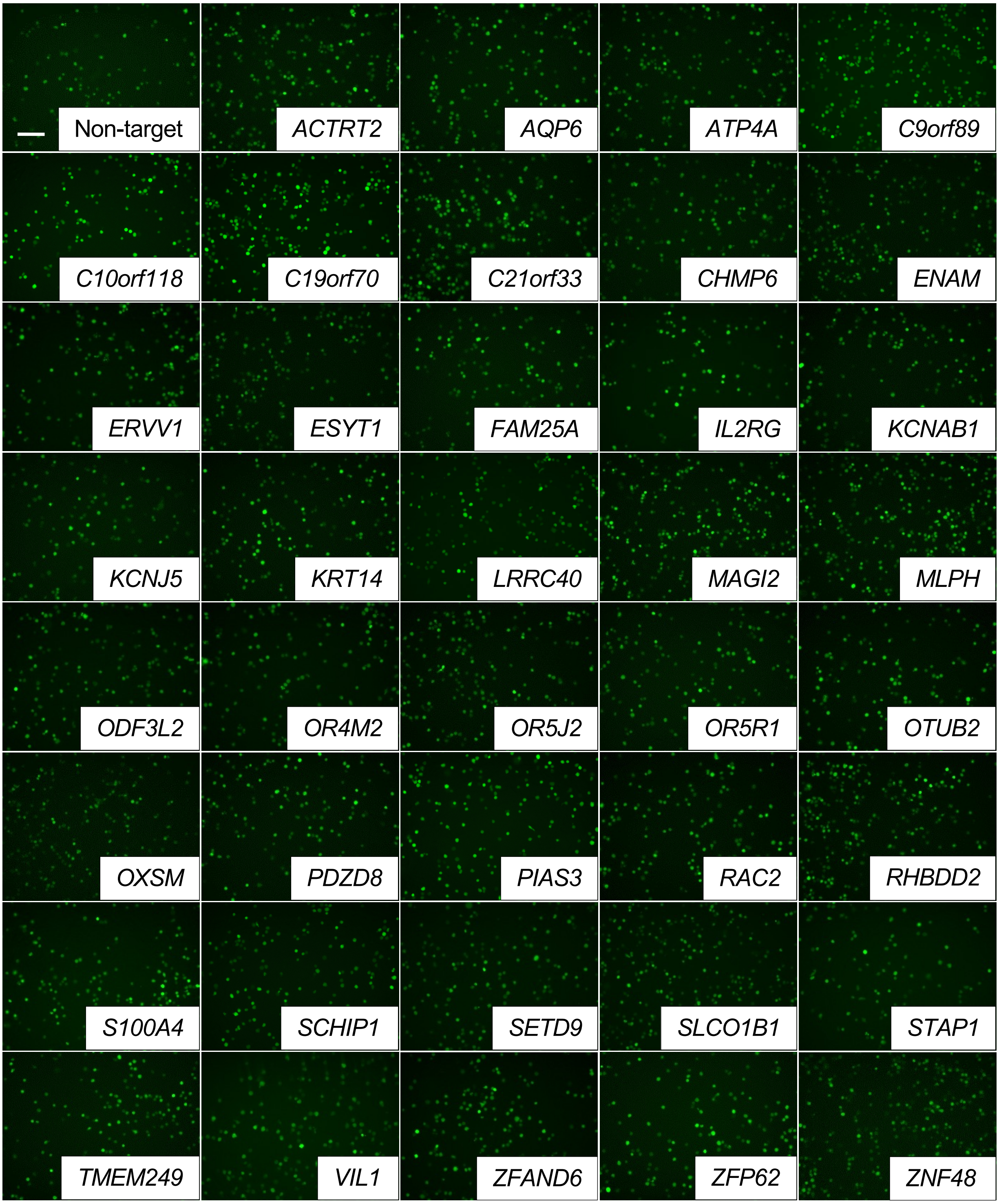

Supplementary Figure 3. Representative images of GFP-positive cells in knockout mutant HEK293T-U937 co-culture experiments for each candidate. GFP-positive cells were indicated as viable U937 cells. After 48 h of exposure to cytarabine, images were captured under a green laser with a 20x objective field of view. Scale bar, 50 μm.

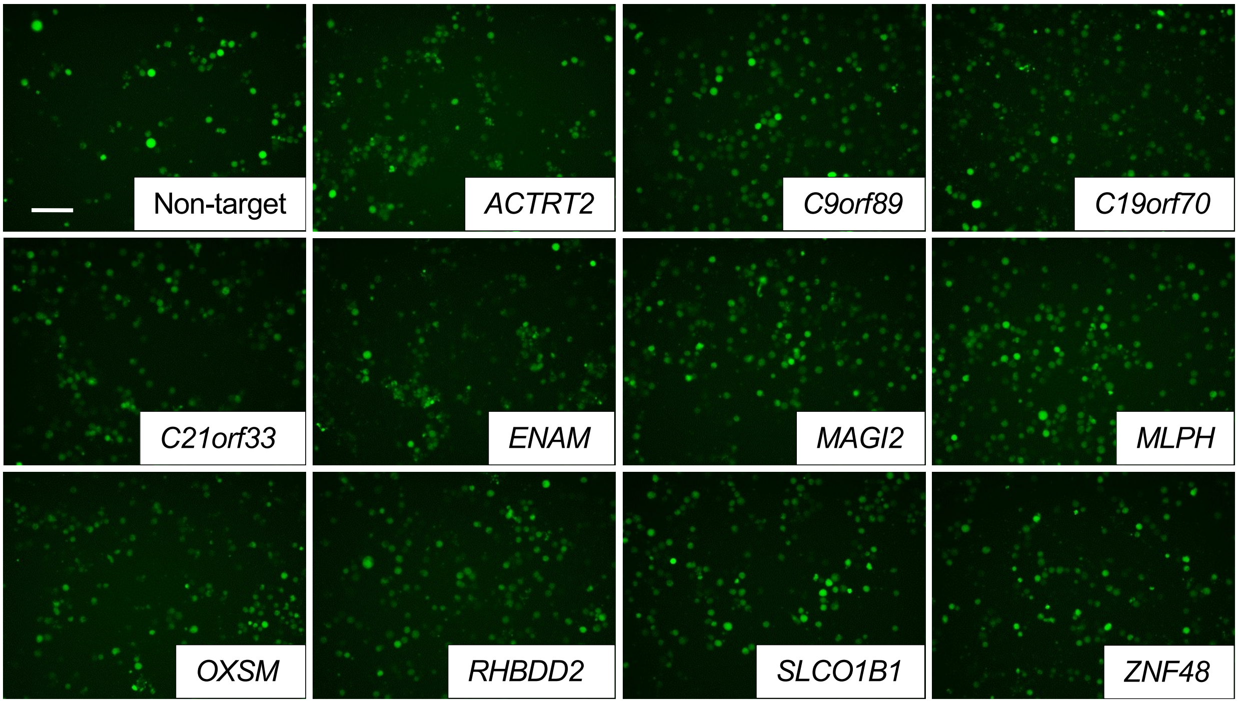

Supplementary Figure 4. Representative images of GFP-positive cells in knockout mutant UE7T-9-U937 co-culture experiments for each candidate. GFP-positive cells were indicated as viable U937 cells. After 48 h of exposure to cytarabine, images were captured under a green laser with a 20x objective field of view. Scale bar, 50 μm.

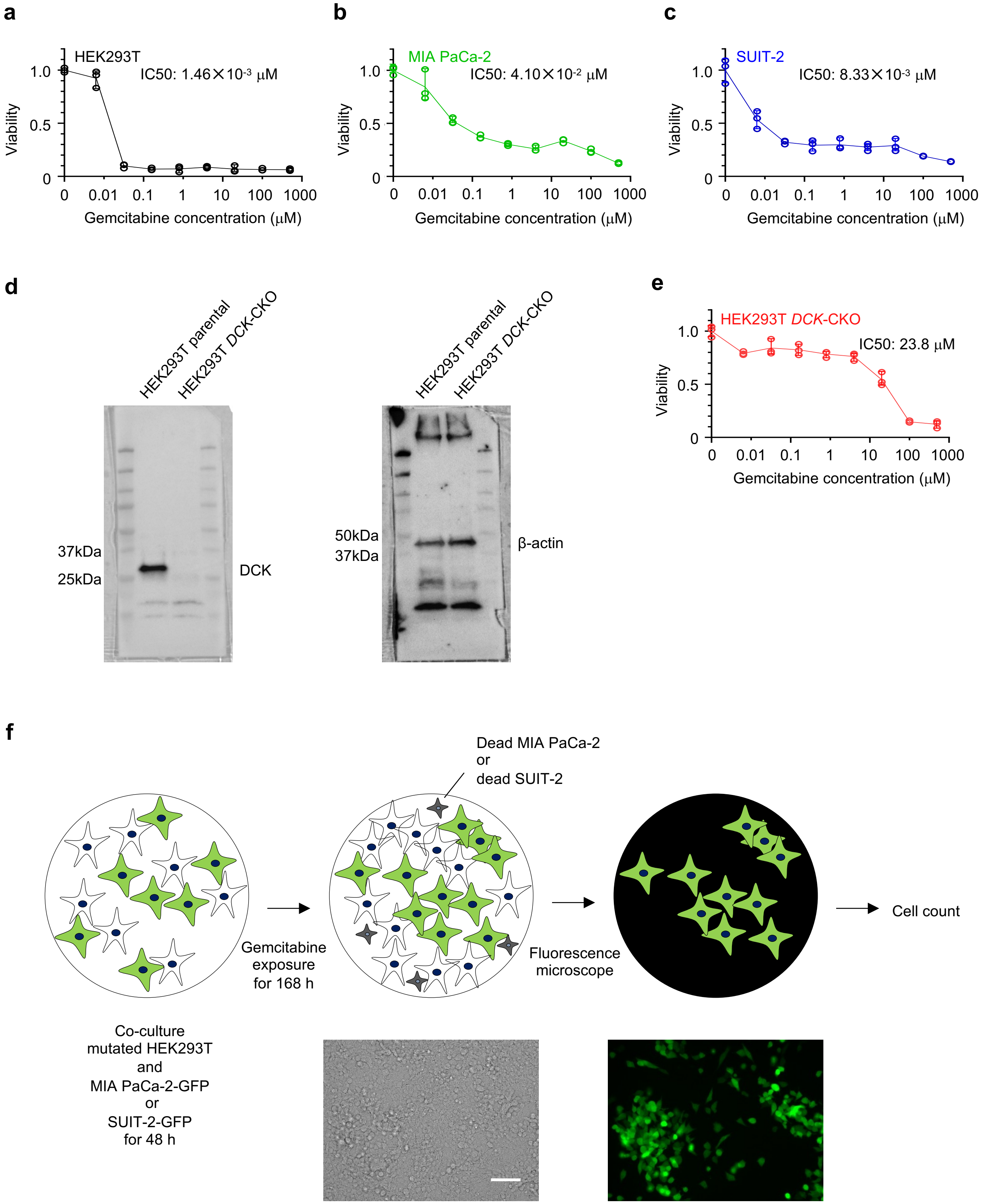

Supplementary Figure 5. Validation experiments to determine drug resistance through cell–cell interactions in HEK293T-pancreatic cancer cell co-culture experiment. (a-c) Dose-response curves of HEK293T (**a**), MIA PaCa-2 (**b**), and SUIT-2 (**c**) cells treated with gemcitabine for 48 h with biological triplication. IC50 to gemcitabine is shown in each graph. **(d)** Loss of DCK in HEK293T *DCK*-CKO clone in the western blot. **(e)** Dose-response curve of HEK293T *DCK*-CKO cells treated with gemcitabine for 48 h with biological triplication. IC50 to gemcitabine is shown in the graph. **(f)** Experimental scheme of HEK293T-pancreatic cancer cell co-culture experiment. Data are represented as mean ± SD. Scale bar, 50 μm.

a

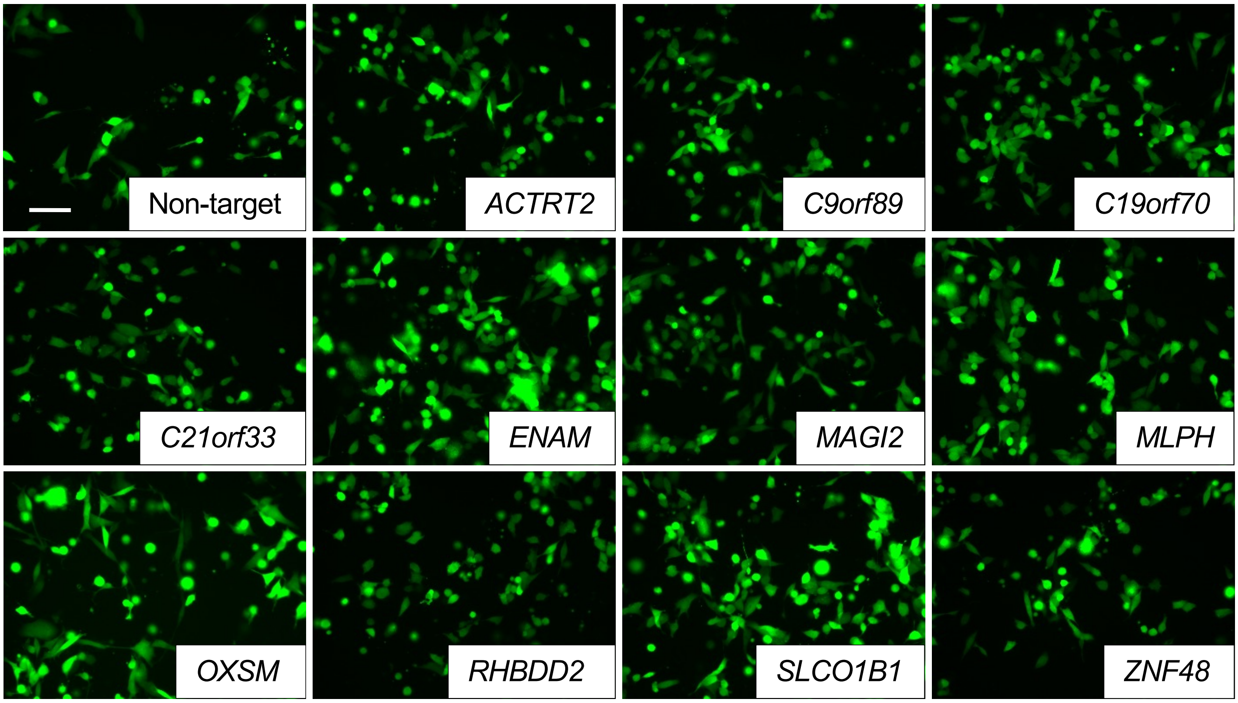

b

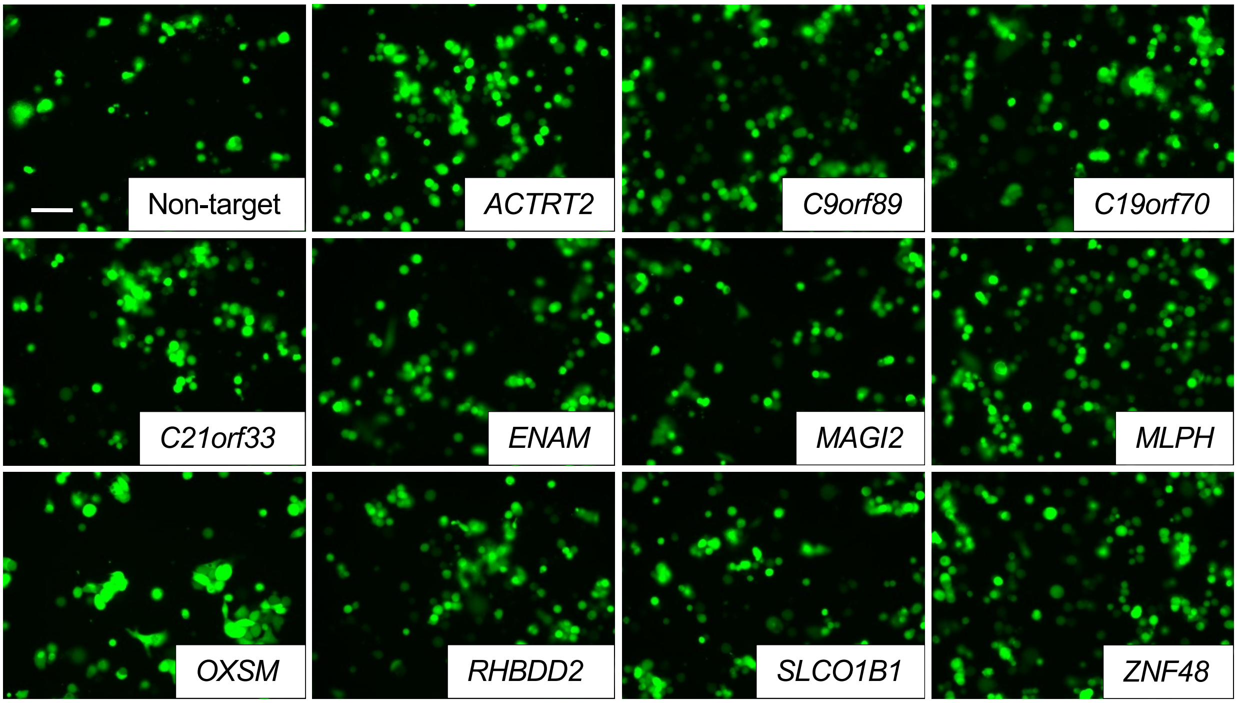

Supplementary Figure 6. Representative images of GFP-positive cells in knockout mutant HEK293T-pancreatic cancer cells co-culture experiments for each candidate. (a, b) GFP-positive cells were indicated as viable pancreatic cancer cells (MIA PaCa-2 (**a**) and SUIT-2 (**b**)). After 168 h of exposure to gemcitabine (10 μM (**a**) or 3 μM (**b**)), images were captured under a green laser with a 20x objective field of view. Scale bar, 50 μm.

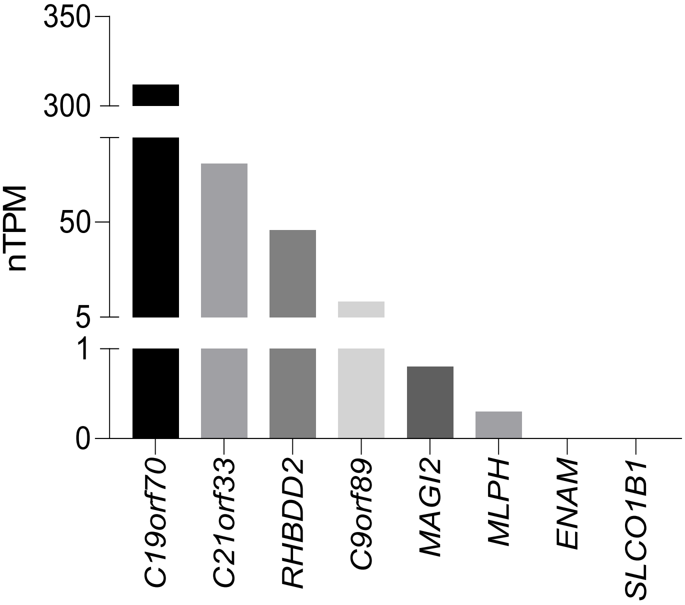

Candidate genes	nTPM
C19orf70	311.9
C21orf33	77.7
RHBDD2	46.3
C9orf89	12.3
MAGI2	0.8
MLPH	0.3
ENAM	0.0
SLCO1B1	0.0

Supplementary Figure 7. Transcriptional product expression of candidate genes in HEK293T. Transcriptional production (nTPM) ≥ 0.1 in HEK293T was defined as high expression based on transcriptional product expression data listed in the cell line RNA database of The Human Protein Atlas(https://www.proteinatlas.org/)[1].

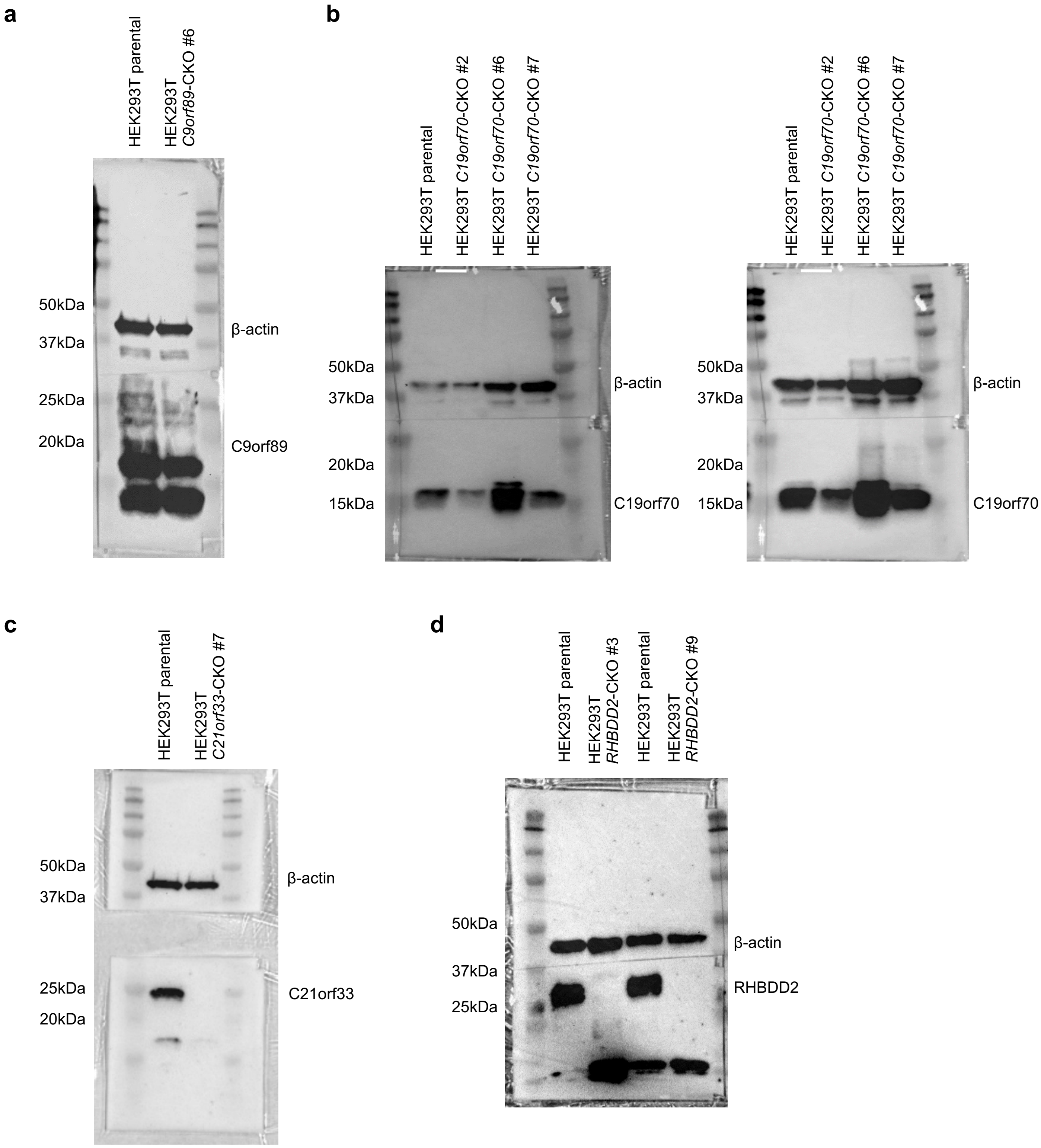

Supplementary Figure 8. Validation of the loss of C9orf89, C19orf70, C21orf33, or RHBDD2 in HEK293T-CKO clones. (a-d) Western blot analysis of C9orf89 (**a**), C19orf70 (**b**), C21orf33 (**c**), or RHBDD2 (**d**) of HEK293T-CKO clones. HEK293T cells of *C9orf89*-CKO #6 (**a**), *C19orf70*-CKO #2 (**b**), *C21orf33*-CKO #7 (**c**), and *RHBDD2*-CKO #9 (**d**) were conducted for the following experiments.

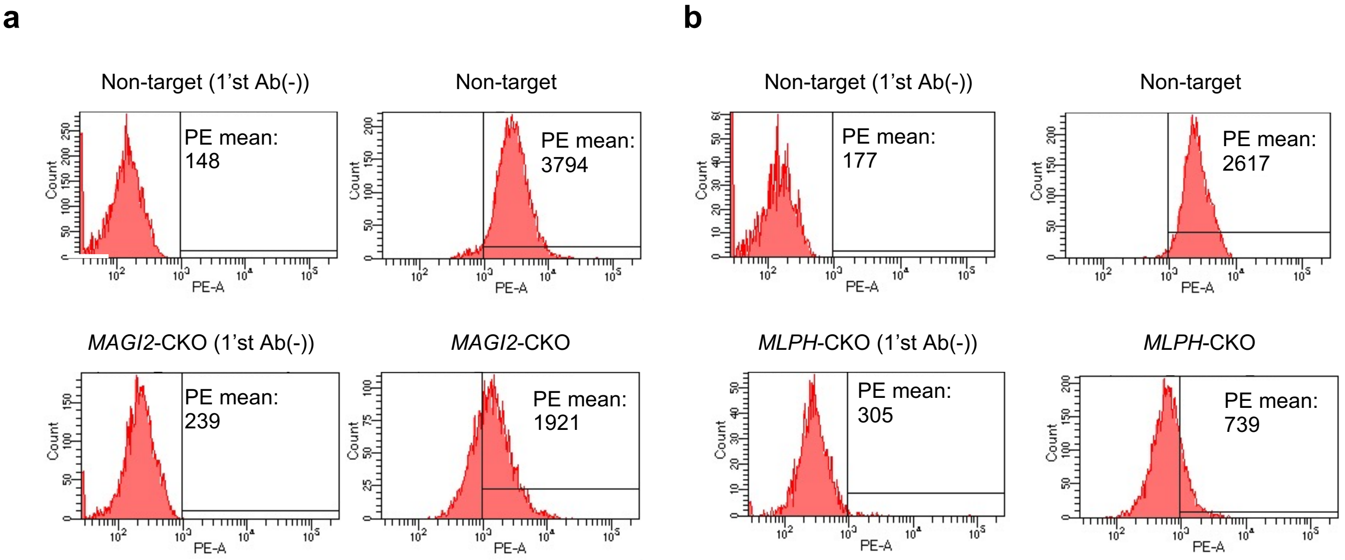

Supplementary Figure 9. Validation of the loss of MAGI2 or MLPH in HEK293T-CKO clones. (a, b) Flow cytometric analysis of the expression of MAGI2 (**a**) or MLPH (**b**) in HEK293T-CKO clones.

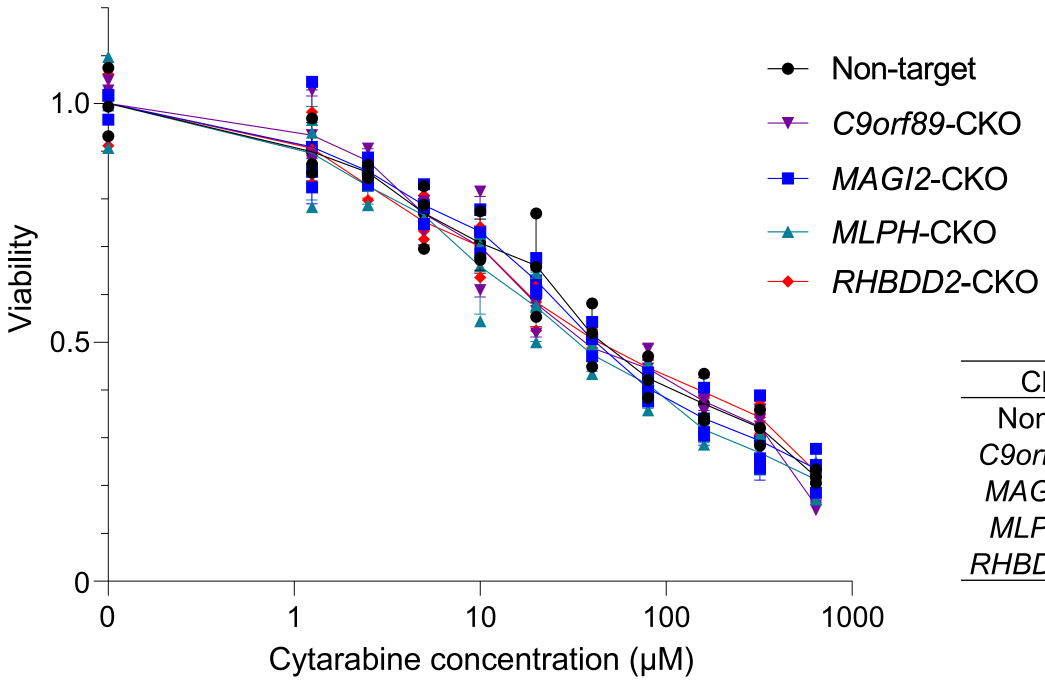

Clones	IC50 (μM)
Non-target	45.3
C9orf89-CKO	36.6
MAGI2-CKO	41.9
MLPH-CKO	33.5
RHBDD2-CKO	43.7

Supplementary Figure 10. Cell viabilities of HEK293T-CKO clones under cytarabine exposure. Dose–response curves for HEK293T cells of *C9orf89*-CKO, *MAGI2*-CKO, *MLPH*-CKO, and *RHBDD2*-CKO, and Non-target treated with cytarabine for 48 h with biological triplication. IC50s to cytarabine are shown. Data are represented as mean ± SD. Statistical significance values were calculated by performing one-way ANOVA with Dunnett's test. No significant differences in viabilities were observed for any concentration of cytarabine.

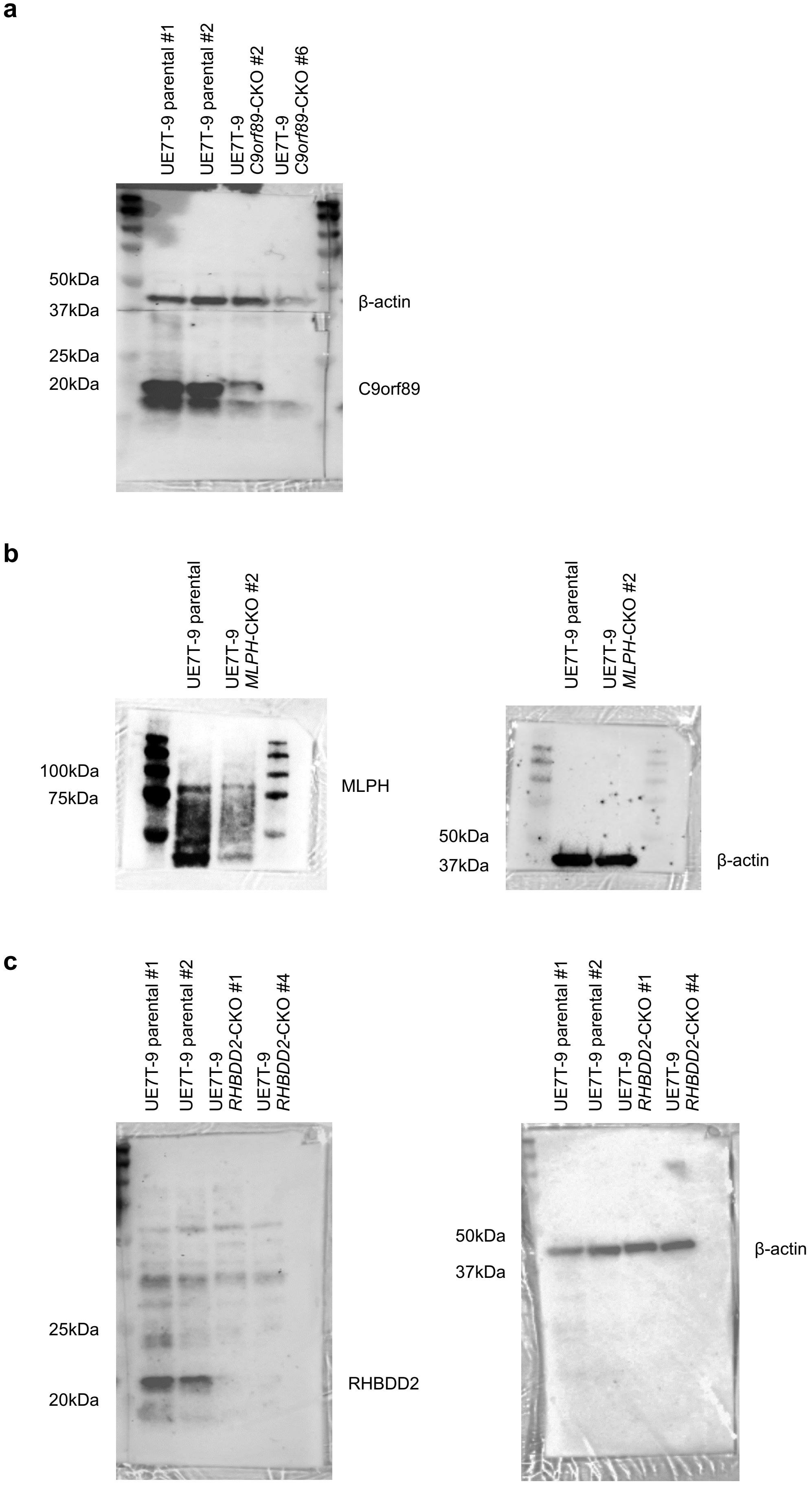

Supplementary Figure 11. Validation of the loss of C9orf89, MLPH, or RHBDD2 in UE7T-9-CKO clones. (a-c) Western blot analysis of C9orf89 (**a**), MLPH(**b**), or RHBDD2 (**c**) of UE7T-9-CKO clones. UE7T-9 cells of *C9orf89*-CKO #2 (**a**), *MLPH*-CKO #2 (**b**), and *RHBDD2*-CKO #1 (**c**) were conducted for the following experiments.

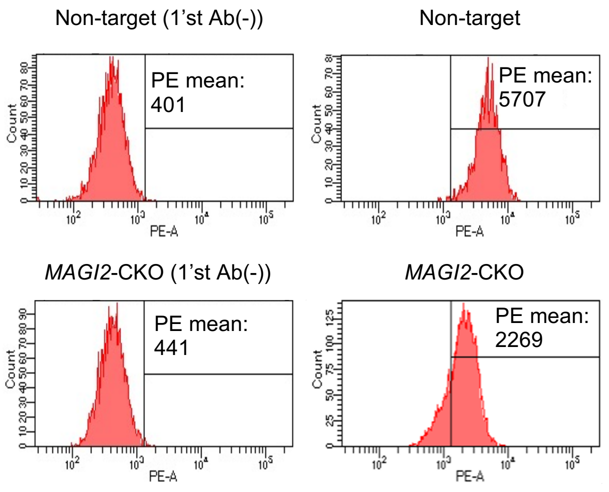

Supplementary Figure 12. Validation of the loss of MAGI2 in UE7T-9 *MAGI2*-CKO. Flow cytometric analysis of the expression of MAGI2 in UE7T-9 *MAGI2*-CKO.

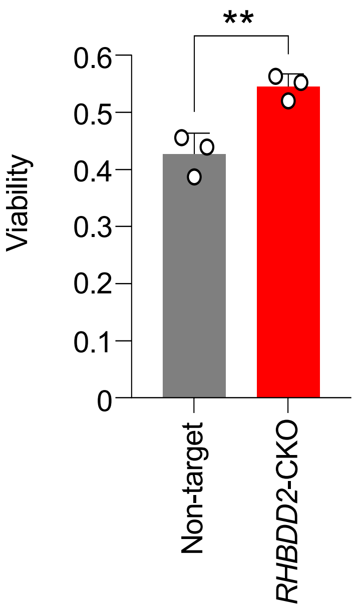

Supplementary Figure 13. Supernatant of HEK293T *RHBDD2*-CKO induced cytarabine resistance in U937. U937 was cultured with the supernatant from HEK293T *RHBDD2*-CKO and treated with 250 nM of cytarabine for 48 h. The viability of U937 cultured with *RHBDD2*-CKO culture supernatant was slightly but significantly increased under cytarabine exposure. The experiment was performed with biological triplication in the three independent experiments. Data are represented as mean ± SD. Statistical significance values were calculated by performing two-tailed unpaired Student's t-tests. **$p < 0.01$.

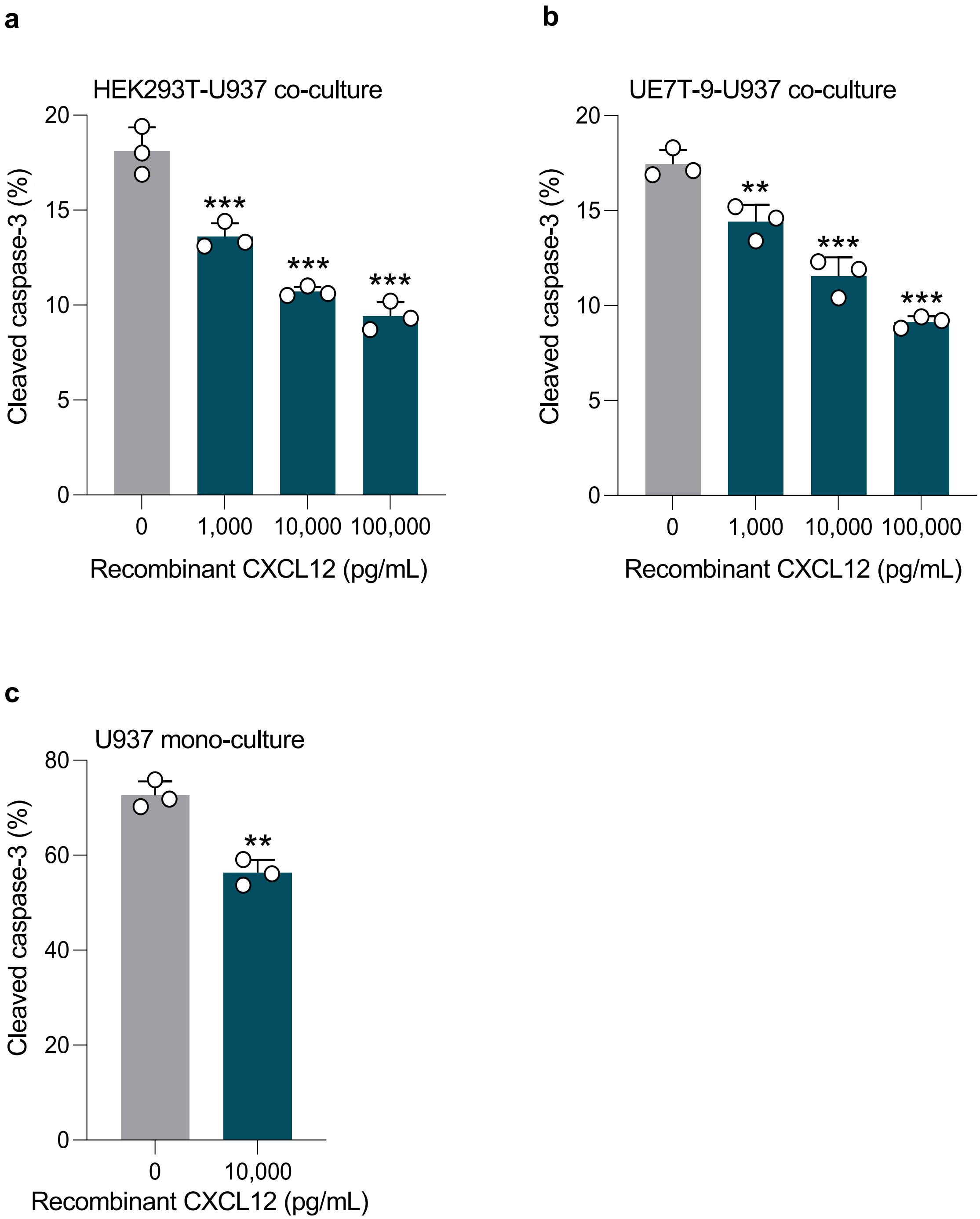

Supplementary Figure 14. Cytarabine exposure experiment with recombinant CXCL12. **(a, b)** HEK293T-U937 and UE7T-9-U937 co-culture experiments with recombinant CXCL12. In the co-culture experiments of HEK293T-U937 (**a**) and UE7T-9-U937 (**b**), pre-treatment with recombinant CXCL12 decreased cleaved caspase-3-positive cells under cytarabine exposure for 48 h. **(c)** U937 mono-culture experiment with recombinant CXCL12. Pre-treatment with recombinant CXCL12 decreased cleaved caspase-3-positive cells under cytarabine exposure for 48 h. The experiment was performed in the two independent experiments. Data are represented as mean ± SD. Statistical significance values were calculated by performing one-way ANOVA with Dunnett's test (**a, b**) or two-tailed unpaired Student's t-tests (**c**). ** $p < 0.01$, ***$p < 0.001$

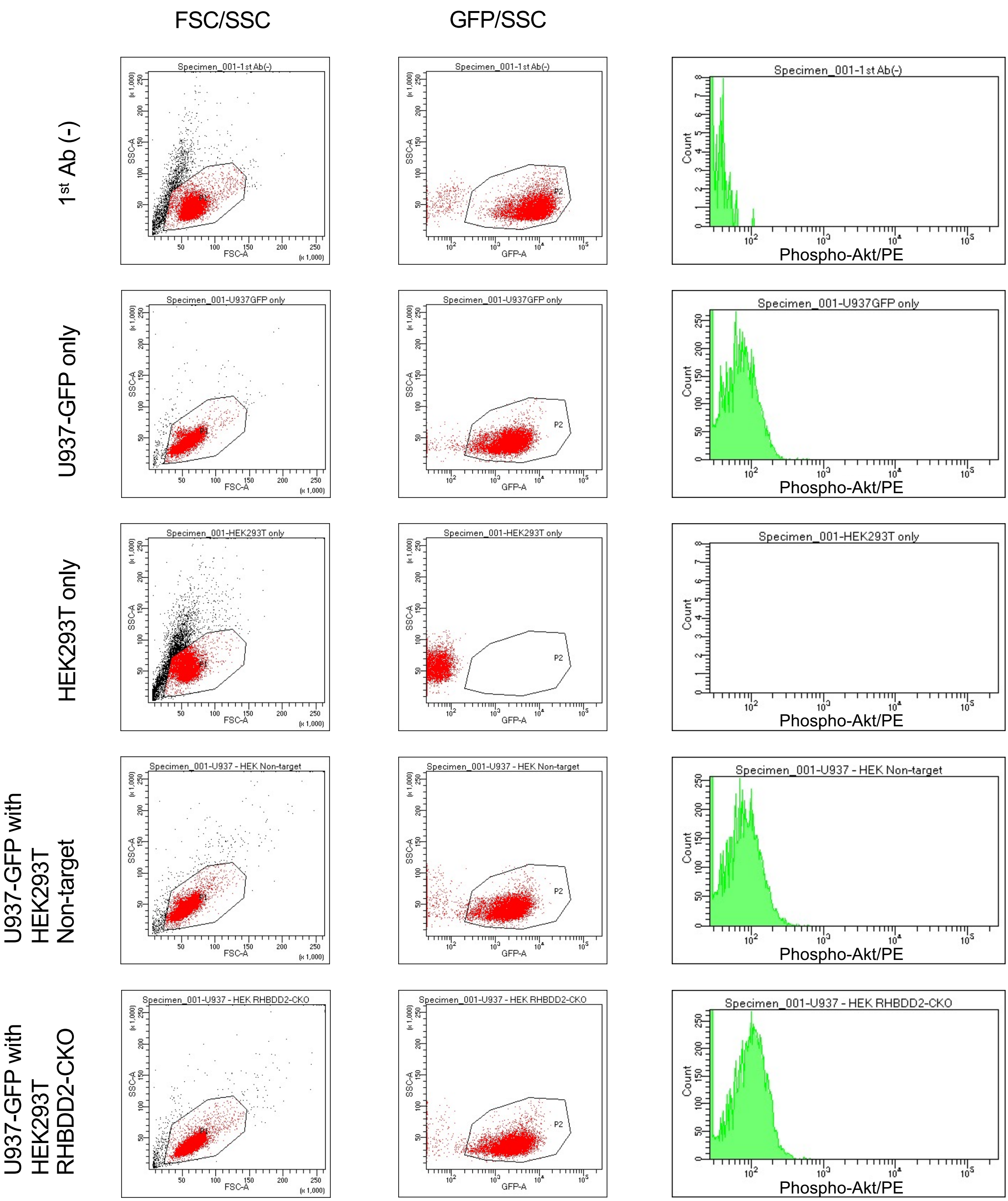

Supplementary Figure 15. Scatter plots of the gating strategy for the analysis of phospho-Akt in the co-culture experiments in U937-GFP-positive cells with HEK293T cells. Expression of phospho-Akt in U937-GFP-positive cells in the present culture system was evaluated by FACSCanto II. The cells were gated based on FSC-A and SSC-A channels to exclude debris and dead cells, and they were further gated based on the GFP-A channel to exclude HEK293T. The mean fluorescence of PE in the GFP-positive population was evaluated as the expression of phospho-Akt in U937-GFP-positive cells.

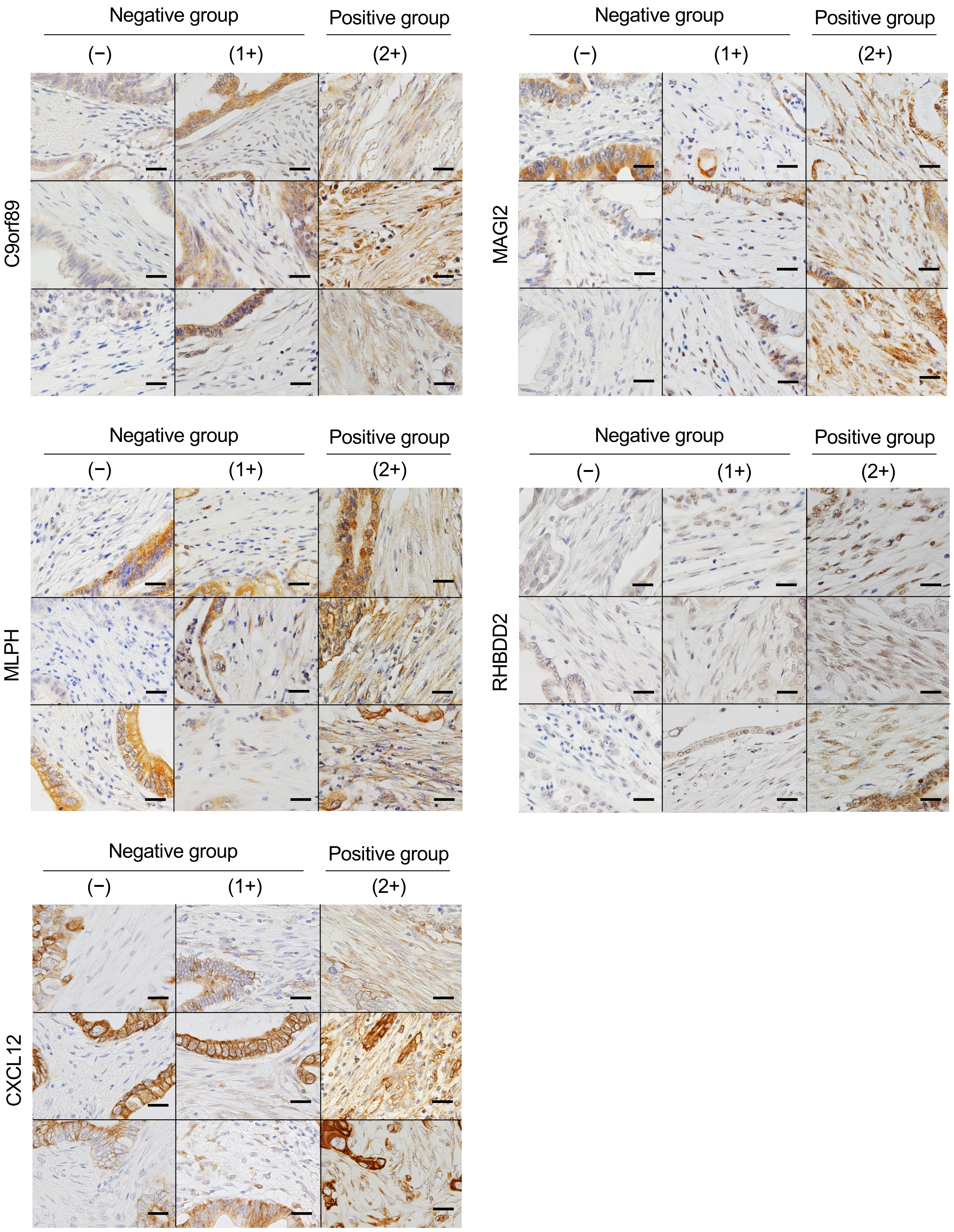

Supplementary Figure 16. Immunostainability score of fibroblasts surrounding pancreatic carcinoma cells. The scoring of immunostainability was assessed based on the following criteria: not stained in fibroblasts was (-), positive in only a few fibroblasts or weakly positive were (1+), and strongly positive on most of the fibroblasts was (2+). Representative images of each antibody are shown. Based on scoring, (-) and (1+) were classified as negative groups, and (2+) was the positive group. Scale bar, 25 μm.

Supplementary Table 1. Guide RNAs in HEK293T clones inducing drug resistance with cell–cell interactions

Library induction	Sequence of gRNA	Gene symbol	Original well ID
1st	TCAGACCTATTGTGACCGCC	*C9orf89*	1-1G12
	ACTGCCGAAGTCTATGCTGC	*KCNAB1*	1-1G12
	TGGTGAGGGAAGTCGCCACA	*VIL1*	1-1G12
	ATGTAGAGTCTAATGCTGAC	*ZFAND6*	1-1G12
	TGACAACCTCTACCTCCGCA	*MAGI2*	1-1G12, 1-8A2
	GGAGACCATATTCGAGAGCC	*ACTRT2*	1-2A5
	GCTCACCTTGCCAGTTGCGA	*RHBDD2*	1-2A5
	AGCCGTCCGAAGGCGAGAGC	*C21orf33*	1-2H9
	TGCTGGCCCGTTTCGAGTGG	*ODF3L2*	1-2H9
	GTAAAGGGACAAACCATGTG	*ERVV-1*	1-4C3
	ATAGATACGACTGCGCCTTT	*SETD9*	1-4C3
	ATCCTGCGAGGGGCTGGTTT	*STAP1*	1-4C3
	GAAATACCTGTTCCATCCGT	*C10orf118*	1-7C12
	GCTCAAGAAGAAGCGATACC	*CHMP6*	1-7C12
	CCTCTTTGCCCGAGTACTTG	*S100A4*	1-7C12
	TATCTACATAGGTTGTTTAA	*SLCO1B1*	1-7C12
	TTATTATTGCCGCTATCCTA	*OR5R1*	1-8A2
	CAATTCTTGTGCTAGACCTG	*LRRC40*	1-8A5
	AAATTGTTGCCTTCACAACC	*OR5J2*	1-8B12
	GGTGAGCGCCCCACTATCTG	*ZNF48*	1-8B12
	CTTGGTGCGGAAGTCATCCG	*KRT14*	1-8H12

Library induction	Sequence of gRNA	Gene symbol	Original well ID
2nd	CACAGACCGTACGCGCCTGC	*KCNJ5*	2-1A1
	TGGCCTATCCCAGACTCCAG	*OR4M2*	2-1A1
	AGTGCGCTTCCTCAAACCGC	*PIAS3*	2-1A1
	TTCAGGATGGTCCCGAAGAA	*OTUB2*	2-1H10
	AACCGGGGGACGTAAGCGCC	*SCHIP1*	2-1H10
	GGCTGGGGGCGCCGTCTACC	*C19orf70*	2-4A3
	TCGAGTACATGAATTGCACT	*IL2RG*	2-4A3
	AGACCCGCTGATCGACTTCG	*PDZD8*	2-4A3, 2-5B7
	GTCTTACCTCCGGGTCCGCC	*MLPH*	2-4H10
	CGATAAGACGATCCCAAACC	*OXSM*	2-4H10
	AAGCCTGCACGCCAACATGC	*AQP6*	2-5B7
	TTGACCAATCTGTCACGCTT	*ATP4A*	2-5B7
	CCCTATGAATGCGACATCTG	*ZFP62*	2-5B7
	CTCCTTCACCACTTCTTCCA	*FAM25A*	2-6F6
	TACTACGTTAATAATGCCGC	*ENAM*	2-7E11
	AATGTTCTTCATCCGACGCC	*ESYT1*	2-7E11
	TTATGAGAACGTCCGCGCCA	*RAC2*	2-7E11
	ACCGGCACGTGGTTCGCCAC	*TMEM249*	2-7E11

Supplementary Table 2.
Univariate and multivariate analyses of factors associated with overall survival in patients with pancreatic ductal carcinoma

Variable	Category	Number of patients	95% CI[a] for MS[b]	*p*-value by log-rank test	HR[c]	95% CI[a] for HR[c]	*p*-value by Cox proportional hazards
Gender	Female	21	23.3-87.0	0.062			
	Male	39	12.9-33.8				
Age	≤ 70y	31	11.7-40.7	0.017	0.54	0.278-1.034	0.063
	> 70y	29	21.1-89.7				
pT[d] category	1–2	10	23.4-NA	0.003	8.67	1.579-47.540	0.013
	3–4	50	13.9-30.2				
Tumor size	≤ 20mm	12	40.7-NA	< 0.001	4.03	0.865-18.780	0.076
	> 20mm	48	12.9-29.4				
UICC stage	I–IIB	44	21.1-86.0	0.001	3.05	1.481-6.284	0.003
	III–IV	16	8.6-24.0				
C9orf89	Positive	40	20.6-66.9	0.089			
	Negative	20	9.4-45.1				
MAGI2	Positive	28	25.6-80.5	0.014	1.187	0.554-2.543	0.659
	Negative	32	12.0-23.6				
MLPH	Positive	25	12.9-66.9	0.514			
	Negative	35	14.4-50.0				
RHBDD2	Positive	36	29.4-86.0	< 0.001	13.59	4.562-40.480	< 0.001
	Negative	24	8.6-16.0				

[a] CI: Confidence interval, [b] MS: Median survival, [c] HR: Hazard ratio, [d] pT: pathological-T.

Supplementary Table 3. Summary of plasmids

Plasmids	Reference
lentiCas9-Blast	Addgene[a], #52962
Human CRISPR Knockout Pooled Library (GeCKO v2)	Addgene[a], #1000000049
lentiCRISPRv2	Addgene[a], #52961
pDendra2-Hygro	Generated with pDendra2-NEO (Takara-Clontech[b], #632545) and sequence for Hygromycin resistant gene
plentiPGK-GFP-pgk-Hygro	Generated wjth plentiPGK-Hygro-DEST (Addgene[a], #19066) and GFP sequence
plentiCMV-GFP-Puro	Generated with plentiCMV-Puro-DEST (Addgene[a], #17452) and GFP sequence
lentiU6-DCK-Hygro	Generated with lentiGuide-puro (Addgene[a], #52963). Sequence for Hygromycin resistant gene was replaced instead of Puromycin resistant gene and integrated target gRNA sequence for DCK

[a] Addgene, MA, USA. [b] Takara-Clontech, Shiga, Japan.

Supplementary Table 4. Summary of primary antibodies

Antibody	Host	Type	Clone	Source	Application	Dilution
C9orf89	mouse	monoclonal	MA5-27443	Thermo[a]	Immunohistochemistry	1:200
					Western Blotting	1:1000
C19orf70	rabbit	polyclonal	LS-C664412	LSBio[b]	Western Blotting	1:1000
C21orf33	rabbit	polyclonal	HPA018517	Sigma-Aldrich[c]	Western Blotting	1:1000
Cleaved Caspase-3	rabbit	monoclonal	9664	Cell Signal[d]	Flow Cytometry	1:6400
CXCL12	mouse	monoclonal	MAB350	R&D[e]	Immunohistochemistry	1:100
DCK	rabbit	polyclonal	HPA062773	Sigma-Aldrich[c]	Western Blotting	1:1000
MAGI2	rabbit	polyclonal	PA5-99245	Thermo[a]	Flow Cytometry	1:200
MAGI2	rabbit	polyclonal	HPA013650	Sigma-Aldrich[c]	Immunohistochemistry	1:200
MLPH	rabbit	polyclonal	10338-1-AP	Proteintech[f]	Flow Cytometry	1:200
					Immunohistochemistry	1:800
					Western Blotting	1:1000
Phospho-Akt	rabbit	monoclonal	4060	Cell Signal[d]	Flow Cytometry	1:100
RHBDD2	rabbit	polyclonal	HPA051960	Sigma-Aldrich[c]	Immunohistochemistry	1:200
					Western Blotting	1:1000

[a] Thermo Fisher Scientific, MA, USA. [b] LifeSpan Biosciences, WA, USA. [c] Sigma-Aldrich, MO, USA.

[d] Cell Signaling Technology, MA, USA. [e] R&D Systems, MN, USA. [f] Proteintech Group, IL, USA.

Supplementary References

1. Uhlén, M. et al. Proteomics. Tissue-based map of the human proteome. *Science* **347**, 1260419 (2015).
