# Supplementary material for: Indirect CRISPR screening with photoconversion revealed key factors of drug resistance with cell–cell interactions
Source: Commun Biol. 2023 Jun 1;6:582. doi: 10.1038/s42003-023-04941-9 (PMC10235018; doi:10.1038/s42003-023-04941-9)
Supplement: Supplementary file 3 — Description of Additional Supplementary Files [file 42003_2023_4941_MOESM3_ESM.pdf]

Description of Additional Supplementary Files

File name: Supplementary Data 1
Description: GSEA analysis data of HEK293T cells of C9orf89-CKO, MAGI2-CKO, MLPHCKO, and RHBDD2-CKO.

File name: Supplementary Data 2
Description: Summary of patient clinical records and immunostainability of peritumoral fibroblasts (n = 60).

File name: Supplementary Data 3
Description: Oligonucleotide sequence.

File name: Supplementary Data 4
Description: The source data behind the graphs in Fig. 1-4.
