# Supplementary material for: Indirect CRISPR screening with photoconversion revealed key factors of drug resistance with cell–cell interactions
Source: Commun Biol. 2023 Jun 1;6:582. doi: 10.1038/s42003-023-04941-9 (PMC10235018; doi:10.1038/s42003-023-04941-9)
Supplement: Supplementary file 8 — Reporting Summary [file 42003_2023_4941_MOESM8_ESM.pdf]

nature portfolio

Corresponding author(s): Morito Kurata

Last updated by author(s): May 13, 2023

Reporting Summary

Nature Portfolio wishes to improve the reproducibility of the work that we publish. This form provides structure for consistency and transparency in reporting. For further information on Nature Portfolio policies, see our Editorial Policies and the Editorial Policy Checklist.

Statistics

For all statistical analyses, confirm that the following items are present in the figure legend, table legend, main text, or Methods section.

n/a	Confirmed	
☐	☒	The exact sample size (*n*) for each experimental group/condition, given as a discrete number and unit of measurement
☐	☒	A statement on whether measurements were taken from distinct samples or whether the same sample was measured repeatedly
☐	☒	The statistical test(s) used AND whether they are one- or two-sided<br>*Only common tests should be described solely by name; describe more complex techniques in the Methods section.*
☒	☐	A description of all covariates tested
☐	☒	A description of any assumptions or corrections, such as tests of normality and adjustment for multiple comparisons
☐	☒	A full description of the statistical parameters including central tendency (e.g. means) or other basic estimates (e.g. regression coefficient) AND variation (e.g. standard deviation) or associated estimates of uncertainty (e.g. confidence intervals)
☐	☒	For null hypothesis testing, the test statistic (e.g. *F*, *t*, *r*) with confidence intervals, effect sizes, degrees of freedom and *P* value noted<br>*Give P values as exact values whenever suitable.*
☒	☐	For Bayesian analysis, information on the choice of priors and Markov chain Monte Carlo settings
☒	☐	For hierarchical and complex designs, identification of the appropriate level for tests and full reporting of outcomes
☒	☐	Estimates of effect sizes (e.g. Cohen's *d*, Pearson's *r*), indicating how they were calculated

Our web collection on statistics for biologists contains articles on many of the points above.

Software and code

Policy information about availability of computer code

Data collection	BD FACS Diva
Data analysis	FlowJo, EZR (version 1.40) and GSEA (version 4.2.0)were used

For manuscripts utilizing custom algorithms or software that are central to the research but not yet described in published literature, software must be made available to editors and reviewers. We strongly encourage code deposition in a community repository (e.g. GitHub). See the Nature Portfolio guidelines for submitting code & software for further information.

Data

Policy information about availability of data

All manuscripts must include a data availability statement. This statement should provide the following information, where applicable:

- Accession codes, unique identifiers, or web links for publicly available datasets
- A description of any restrictions on data availability
- For clinical datasets or third party data, please ensure that the statement adheres to our policy

Source data used in Fig. 2-4 are provided in Supplementary Data 4. Source data of Fig. 5b were provided in Supplementary Data 2. All other data generated, including raw data supporting, in this study have been deposited in figshare.com.
Fig. 1 (https://figshare.com/s/3092ec8b6e9698f7d2a7),
Supplementary Fig. 5 (https://figshare.com/s/ec9e74e885b91d41dcef),

Supplementary Fig. 8, 9, 11, 12 (https://figshare.com/s/ce402d008054d7d9a333),
plasmid maps (https://figshare.com/s/b9c98a0206a6972ea0ed),
and all other revised raw data (10.6084/m9.figshare.22492372).
RNA-seq data have been deposited in Gene Expression Omnibus (GEO) under access number GSE 203256. Following plasmids have been deposited; pDendra2-Hygro (Addgene ID:#202407) and pLenti-DCK-Hygro (Addgene ID:#202409).

Human research participants

Policy information about studies involving human research participants and Sex and Gender in Research.

Reporting on sex and gender	The ratio of Females / Males is 21 / 39.
Population characteristics	Sixty clinical samples of invasive pancreatic ductal carcinoma were obtained from 60 patients who underwent operations without neoadjuvant chemotherapy at the Tokyo Medical and Dental University Hospital, Tokyo between 2008 and 2016. The following five parameters were evaluated as clinicopathologic factors: sex (female vs. male), age (≤ 70 y vs. > 70 y), pT category (1–2 vs. 3–4), tumor size (≤ 20 mm vs. > 20 mm), and UICC stage (I–IIB vs. III–IV).
Recruitment	This study is a retrospective study. The 60 patients samples were used, who had invasive pancreatic ductal carcinoma and underwent operations without neoadjuvant chemotherapy at the Tokyo Medical and Dental University Hospital, Tokyo, between 2008 and 2016.
Ethics oversight	Informed consent was obtained from all patients by an opt-out method. The study was approved by the ethics committees of Tokyo Medical and Dental University. All procedures were performed following the ethical standards established by these committees (M2000-1458-07).

Note that full information on the approval of the study protocol must also be provided in the manuscript.

Field-specific reporting

Please select the one below that is the best fit for your research. If you are not sure, read the appropriate sections before making your selection.

☒ Life sciences ☐ Behavioural & social sciences ☐ Ecological, evolutionary & environmental sciences

For a reference copy of the document with all sections, see nature.com/documents/nr-reporting-summary-flat.pdf

Life sciences study design

All studies must disclose on these points even when the disclosure is negative.

Sample size	For each co-culture experiment, three wells were prepared with the same conditions, and three fields were randomly captured for each well with the drug exposure.<br>In the other in-vitro experiments , the results were obtained independently in triplicate for each experiment.<br>In the analysis of clinical samples, 60 clinical samples from 60 patients who underwent surgical operations without neoadjuvant chemotherapy at the Tokyo Medical and Dental University Hospital, Tokyo, between 2008 and 2016 were used.
Data exclusions	No data were excluded.
Replication	The results were obtained independently in triplicate for each experiment, and the experiments were usually repeated two or three times.
Randomization	For each co-culture experiment, three wells were prepared with the same conditions, and three fields were randomly captured for each well with the drug exposure.
Blinding	For each co-culture experiment, the observers were blinded to sample identity and selected observation fields of view randomly. The count of viable cells per field of view was performed by different blinded observers from observers capturing images using Image J.<br>For the scoring of immunostainability of clinical samples, scoring was performed by different blinded pathologists (K. Sugita and M. Kurata) from observers performing immunostaining .

Reporting for specific materials, systems and methods

We require information from authors about some types of materials, experimental systems and methods used in many studies. Here, indicate whether each material, system or method listed is relevant to your study. If you are not sure if a list item applies to your research, read the appropriate section before selecting a response.

Materials & experimental systems

n/a	Involved in the study	
☐	☒	Antibodies
☐	☒	Eukaryotic cell lines
☒	☐	Palaeontology and archaeology
☒	☐	Animals and other organisms
☐	☒	Clinical data
☒	☐	Dual use research of concern

Methods

n/a	Involved in the study	
☒	☐	ChIP-seq
☐	☒	Flow cytometry
☒	☐	MRI-based neuroimaging

Antibodies

Antibodies used

Primary antibodies
Anti-C9orf89, mouse, monoclonal, MA5-27443, Thermo Fisher Scientific.
Anti-C19orf70, rabbit, polyclonal , LS-C664412, LifeSpan Biosciences.
Anti-C21orf33, rabbit, polyclonal, HPA018517, Sigma-Aldrich.
Anti-Cleaved Caspase-3, rabbit, monoclonal, 9664, Cell Signaling Technology.
Anti-CXCL12, mouse, monoclonal, MAB350, R&D Systems.
Anti-DCK, rabbit, polyclonal, HPA062773, Sigma-Aldrich.
Anti-MAGI2, rabbit, polyclonal, PA5-99245, Thermo Fisher Scientific.
Anti-MAGI2, rabbit, polyclonal, HPA013650, Sigma-Aldrich.
Anti-MLPH, rabbit, polyclonal, 10338-1-AP, Proteintech Group.
Anti-Phospho-Akt, rabbit, monoclonal, 4060, Cell Signaling Technology.
Anti-RHBDD2, rabbit, polyclonal, HPA051960, Sigma-Aldrich.

Secondary antibody
Anti-rabbit IgG (H+L), F(ab')2 Fragment (PE conjugate), 4412, Cell Signaling Technology.

Validation

No validation for commercial antibodies.

Eukaryotic cell lines

Policy information about cell lines and Sex and Gender in Research

Cell line source(s)

HEK293T, UE7T-9, MIA PaCa-2, and SUIT-2 cells were obtained from the JCRB Cell Bank (National Institutes of Biomedical Innovation, Health and Nutrition, JAPAN).

Authentication

The authentication of U937 cells was confirmed using a 10-loci multiplex short tandem repeat analysis provided by the cell authentication services of BEX Co., LTD (Japan). Other cells were used for experiments immediately after obtain.

Mycoplasma contamination

U937 tested negative for mycoplasma contamination and was frozen as aliquots and each vial was used for experiments immediately after thawing. All other cell lines were purchased by the vendor and immediately frozen as aliquots and each vial was used for the experiments immediately after thawing. Therefore, the mycoplasma contamination test is not performed.

Commonly misidentified lines
(See ICLAC register)

No commonly misidentified cell lines were used.

Clinical data

Policy information about clinical studies
All manuscripts should comply with the ICMJE guidelines for publication of clinical research and a completed CONSORT checklist must be included with all submissions.

Clinical trial registration

N.A.

Study protocol

N.A.

Data collection

This study is a retrospective study and contain the result of "Tumor marker prognostic studies". The 60 patients samples were used, who had invasive pancreatic ductal carcinoma and underwent operations without neoadjuvant chemotherapy at the Tokyo Medical and Dental University Hospital, Tokyo between 2008 and 2016.

Outcomes

The immunohistochemical expression of candidates in our Indirect CRISPR screenings might suggest drug resistance (poor prognosis) in pancreatic cancer.

Flow Cytometry

Plots

Confirm that:

☒ The axis labels state the marker and fluorochrome used (e.g. CD4-FITC).

☒ The axis scales are clearly visible. Include numbers along axes only for bottom left plot of group (a 'group' is an analysis of identical markers).

☒ All plots are contour plots with outliers or pseudocolor plots.

☒ A numerical value for number of cells or percentage (with statistics) is provided.

Methodology

Sample preparation	Cell suspensions were used.
Instrument	BD FACSCantoII and BD FACSAriaIII (Becton Dickinson and Company).
Software	FlowJo
Cell population abundance	In the sorting of the Dendra2 photoconverted HEK293T cells, approximately 19,000 photoconverted cells were sorted.<br>In the analysis of cleaved caspase-3 and phospho-Akt, 10,000 cells were measured per each sample.
Gating strategy	In the sorting of the Dendra2 photoconverted HEK293T cells, the cells were initially gated based on FSC-A and SSC-A channels to exclude the debris and dead cells. Subsequently, the PI channel-positive cells were sorted as the populations that exhibited negligible signals in the un-photoconverted negative controls. Approximately 1,000 photoconverted cells were sorted per each well of the cells in the wells that underwent photoconversion.<br>In the analysis of cleaved caspase-3, the cells were gated based on PE-A and SSC-A channels as populations that exhibited negligible signals in the unstained negative controls.<br>In the analysis of phospho-Akt, the cells were gated based on FSC-A and SSC-A channels to exclude debris and dead cells, and they were further gated based on the GFP-A channel to exclude HEK293T/UE7T-9,. Subsequently, the cells were gated based on the PE-A channel as the populations that exhibited negligible signals in the unstained negative controls.

☐ Tick this box to confirm that a figure exemplifying the gating strategy is provided in the Supplementary Information.
